# 3-Iodo-2-methyl-1-phenyl­sulfonyl-1*H*-indole

**DOI:** 10.1107/S1600536811004685

**Published:** 2011-02-12

**Authors:** C. Ramathilagam, Velu Saravanan, A. K. Mohanakrishnan, G. Chakkaravarthi, P. R. Umarani, V. Manivannan

**Affiliations:** a Department of Physics, AMET University, Kanathur, Chennai 603 112, India; b Department of Organic Chemistry, University of Madras, Guindy Campus, Chennai 600 025, India; c Department of Physics, CPCL Polytechnic College, Chennai 600 068, India; d Department of Physics, Presidency College (Autonomous), Chennai 600 005, India; e Department of Research and Development, PRIST University, Vallam, Thanjavur 613 403, Tamil Nadu, India

## Abstract

In the title compound, C_15_H_12_INO_2_S, the sulfonyl-bound phenyl ring forms a dihedral angle 82.84 (9)° with the indole ring system. The mol­ecular structure is stabilized by a weak intra­molecular C—H⋯O hydrogen bond. The crystal structure exhibits weak inter­molecular C—H⋯π inter­actions and π–π inter­actions between the indole groups [centroid–centroid distance between the five-membered and six-membered rings of the indole group = 3.7617 (18) Å].

## Related literature

For the biological properties of indole derivatives, see: Chai *et al.* (2006 ▶); Nieto *et al.* (2005 ▶). For the structures of closely related compounds, see: Chakkaravarthi *et al.* (2007 ▶, 2008 ▶).
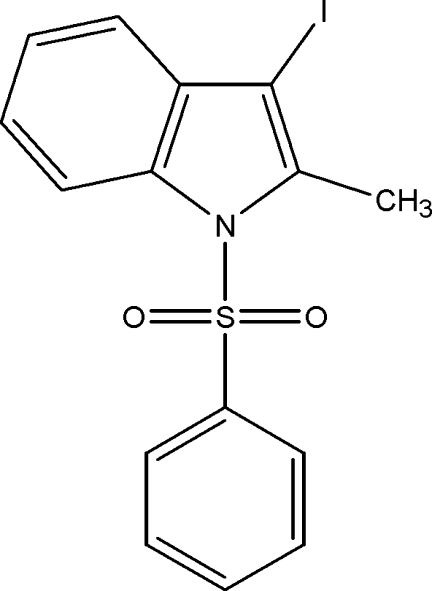


## Experimental

### 

#### Crystal data

C_15_H_12_INO_2_S
*M* *_r_* = 397.22
Monoclinic, 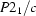

*a* = 10.7068 (3) Å
*b* = 16.2670 (4) Å
*c* = 8.5147 (2) Å
β = 104.540 (1)°
*V* = 1435.49 (6) Å^3^
*Z* = 4
Mo *K*α radiation
μ = 2.38 mm^−1^
*T* = 295 K
0.30 × 0.24 × 0.20 mm

#### Data collection

Bruker Kappa APEXII diffractometer
Absorption correction: multi-scan (*SADABS*; Sheldrick, 1996 ▶) *T*_min_ = 0.536, *T*_max_ = 0.648
21249 measured reflections
5276 independent reflections
3696 reflections with *I* > 2σ(*I*)
*R*_int_ = 0.023

#### Refinement

*R*[*F* ^2^ > 2σ(*F* ^2^)] = 0.043
*wR*(*F* ^2^) = 0.144
*S* = 1.06
5276 reflections
182 parameters
1 restraint
H-atom parameters constrained
Δρ_max_ = 0.94 e Å^−3^
Δρ_min_ = −1.56 e Å^−3^

### 

Data collection: *APEX2* (Bruker, 2004 ▶); cell refinement: *SAINT* (Bruker, 2004 ▶); data reduction: *SAINT*; program(s) used to solve structure: *SHELXS97* (Sheldrick, 2008 ▶); program(s) used to refine structure: *SHELXL97* (Sheldrick, 2008 ▶); molecular graphics: *PLATON* (Spek, 2009 ▶); software used to prepare material for publication: *SHELXL97*.

## Supplementary Material

Crystal structure: contains datablocks I, global. DOI: 10.1107/S1600536811004685/gk2346sup1.cif

Structure factors: contains datablocks I. DOI: 10.1107/S1600536811004685/gk2346Isup2.hkl

Additional supplementary materials:  crystallographic information; 3D view; checkCIF report

## Figures and Tables

**Table 1:** Hydrogen-bond geometry (Å, °) *Cg*3 is the centroid of the C9–C14 ring.

*D*—H⋯*A*	*D*—H	H⋯*A*	*D*⋯*A*	*D*—H⋯*A*
C13—H13⋯O1	0.93	2.29	2.871 (4)	120
C4—H4⋯*Cg*3^i^	0.93	2.65	3.517 (5)	156

Symmetry code: (i) 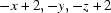.

**Fig. 1.**
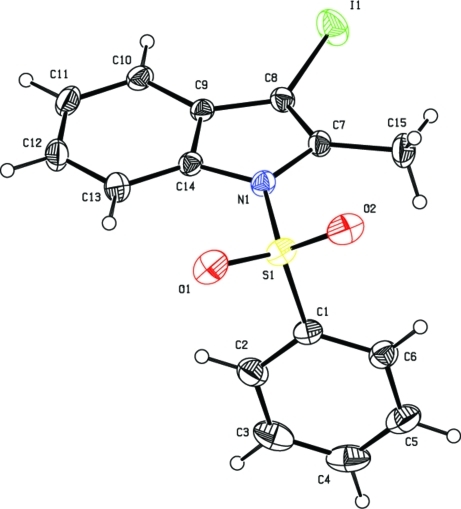
The molecular structure of the title compound with 30% probability displacement ellipsoids for non-H atoms.

**Fig. 2.**
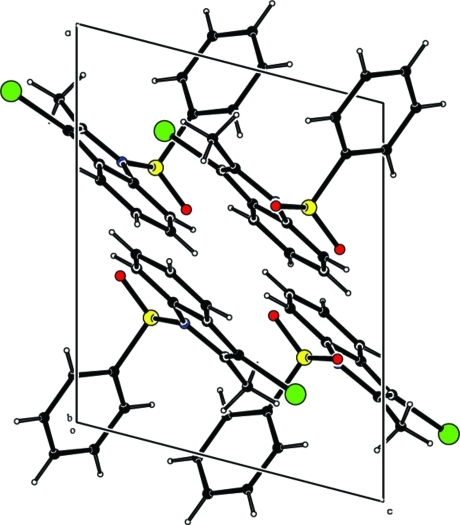
Crystal packing viewed along the b axis.

## supplementary crystallographic information

### Comment

Indole derivatives exhibit antihepatitis B virus (Chai *et al.*, 2006) and antibacterial (Nieto *et al.*, 2005) activities. The geometric parameters of the title molecule (Fig. 1) agree well with the reported similar structures (Chakkaravarthi *et al.* 2007, 2008).

The phenyl ring makes the dihedral angle of 82.84 (9)° with the indole ring system. The sum of the bond angles around N1 [359.4 (2)°] indicates that N1 atom is *sp*^2^ hybridized. The molecular structure is stabilized by weak intramolecular C—H···O hydrogen bond. The crystal structure exhibits weak intermolecular C—H···π (Table 1) and π–π interactions [*Cg*1···*Cg*3 (1 - *x*,-*y*,1 - *z*) 3.7617 (18) Å; *Cg*1 and *Cg*3 are the centroids of the rings N1/C7/C8/C9/C14 and C9—C14, respectively].

### Experimental

3-Iodo-2-methylindole (5 g,0.02 mmole) was dissolved in distilled benzene (100 ml).To this, benzenesulfonyl chloride(3.23 ml,0.025 mmol) and 60% aqueous NaOH solution (40 g in 67.0 ml) were added along with tetrabutyl ammonium hydrogensulfate (1.0 g). This two phase system was stirred at room temperature for 2 h. It was then diluted with water (200 ml) and the organic layer was separated. The aqueous layer was extracted with benzene (2x20 ml). The combined organic layer was dried(Na_2_SO_4_).The benzene was then removed completely and the crude product was recrystallized from methanol (m.p. 395–397 K).

### Refinement

H atoms were positioned geometrically and refined using riding model with C—H = 0.93 Å and *U*_iso_(H) = 1.2Ueq(C) for aromatic C—H and C—H = 0.96 Å and *U*_iso_(H) = 1.5Ueq(C) for CH_3_. The components of the anisotropic displacement parameters in direction of the bond of I1 and C8 were restrained to be equal within an effective standard deviation of 0.001 using the DELU command in *SHELXL* (Sheldrick, 2008).

### Crystal data

**Table:**

C_15_H_12_INO_2_S	*F*(000) = 776
*M**_r_* = 397.22	*D*_x_ = 1.838 Mg m^−^^3^
Monoclinic, *P*2_1_/*c*	Mo *K*α radiation, λ = 0.71073 Å
Hall symbol: -P 2ybc	Cell parameters from 8510 reflections
*a* = 10.7068 (3) Å	θ = 2.5–30.4°
*b* = 16.2670 (4) Å	µ = 2.38 mm^−^^1^
*c* = 8.5147 (2) Å	*T* = 295 K
β = 104.540 (1)°	Block, colourless
*V* = 1435.49 (6) Å^3^	0.30 × 0.24 × 0.20 mm
*Z* = 4	

### Data collection

**Table:**

Bruker Kappa APEXII diffractometer	5276 independent reflections
Radiation source: fine-focus sealed tube	3696 reflections with *I* > 2σ(*I*)
graphite	*R*_int_ = 0.023
ω and φ scans	θ_max_ = 32.8°, θ_min_ = 2.5°
Absorption correction: multi-scan (*SADABS*; Sheldrick, 1996)	*h* = −15→16
*T*_min_ = 0.536, *T*_max_ = 0.648	*k* = −23→24
21249 measured reflections	*l* = −12→11

### Refinement

**Table:**

Refinement on *F*^2^	Primary atom site location: structure-invariant direct methods
Least-squares matrix: full	Secondary atom site location: difference Fourier map
*R*[*F*^2^ > 2σ(*F*^2^)] = 0.043	Hydrogen site location: inferred from neighbouring sites
*wR*(*F*^2^) = 0.144	H-atom parameters constrained
*S* = 1.06	*w* = 1/[σ^2^(*F*_o_^2^) + (0.0743*P*)^2^ + 0.987*P*] where *P* = (*F*_o_^2^ + 2*F*_c_^2^)/3
5276 reflections	(Δ/σ)_max_ < 0.001
182 parameters	Δρ_max_ = 0.94 e Å^−^^3^
1 restraint	Δρ_min_ = −1.56 e Å^−^^3^

### Fractional atomic coordinates and isotropic or equivalent isotropic displacement parameters (Å^2^)

**Table:**

	*x*	*y*	*z*	*U*_iso_*/*U*_eq_	
C1	0.8489 (3)	0.14775 (16)	0.8788 (3)	0.0350 (5)	
C2	0.8848 (4)	0.0951 (2)	1.0097 (4)	0.0495 (7)	
H2	0.8265	0.0574	1.0324	0.059*	
C3	1.0098 (4)	0.0998 (3)	1.1063 (5)	0.0638 (10)	
H3	1.0365	0.0646	1.1944	0.077*	
C4	1.0940 (4)	0.1562 (3)	1.0722 (5)	0.0643 (11)	
H4	1.1778	0.1587	1.1375	0.077*	
C5	1.0569 (3)	0.2092 (3)	0.9431 (5)	0.0556 (9)	
H5	1.1149	0.2478	0.9227	0.067*	
C6	0.9331 (3)	0.20492 (19)	0.8436 (4)	0.0433 (6)	
H6	0.9073	0.2398	0.7549	0.052*	
C7	0.7397 (3)	0.04833 (16)	0.5161 (3)	0.0329 (5)	
C8	0.7262 (3)	−0.03190 (16)	0.4724 (3)	0.0336 (5)	
C9	0.6619 (2)	−0.07527 (14)	0.5739 (3)	0.0297 (4)	
C10	0.6230 (3)	−0.15718 (17)	0.5777 (4)	0.0387 (6)	
H10	0.6379	−0.1950	0.5025	0.046*	
C11	0.5626 (3)	−0.1805 (2)	0.6940 (4)	0.0478 (7)	
H11	0.5362	−0.2348	0.6978	0.057*	
C12	0.5402 (3)	−0.1249 (2)	0.8061 (4)	0.0486 (7)	
H12	0.5005	−0.1429	0.8852	0.058*	
C13	0.5753 (3)	−0.0428 (2)	0.8043 (4)	0.0435 (6)	
H13	0.5585	−0.0055	0.8792	0.052*	
C14	0.6367 (2)	−0.01869 (15)	0.6857 (3)	0.0309 (5)	
C15	0.7993 (4)	0.1168 (2)	0.4432 (5)	0.0509 (7)	
H15A	0.8230	0.0972	0.3483	0.076*	
H15B	0.8748	0.1362	0.5208	0.076*	
H15C	0.7383	0.1609	0.4137	0.076*	
N1	0.6822 (2)	0.05838 (13)	0.6481 (3)	0.0335 (4)	
O1	0.6046 (2)	0.13114 (15)	0.8577 (4)	0.0552 (6)	
O2	0.6719 (2)	0.21172 (13)	0.6496 (3)	0.0533 (6)	
S1	0.68958 (6)	0.14402 (4)	0.75748 (10)	0.03797 (16)	
I1	0.78752 (3)	−0.084909 (16)	0.28650 (3)	0.06383 (12)	

### Atomic displacement parameters (Å^2^)

**Table:**

	*U*^11^	*U*^22^	*U*^33^	*U*^12^	*U*^13^	*U*^23^
C1	0.0355 (12)	0.0307 (11)	0.0379 (13)	0.0043 (9)	0.0078 (10)	−0.0078 (10)
C2	0.0583 (19)	0.0487 (17)	0.0391 (15)	0.0014 (14)	0.0076 (14)	0.0020 (12)
C3	0.071 (3)	0.070 (2)	0.0421 (18)	0.020 (2)	−0.0008 (17)	0.0018 (17)
C4	0.0421 (17)	0.087 (3)	0.056 (2)	0.0121 (17)	−0.0013 (15)	−0.025 (2)
C5	0.0409 (16)	0.065 (2)	0.061 (2)	−0.0088 (14)	0.0131 (14)	−0.0205 (18)
C6	0.0436 (15)	0.0406 (14)	0.0458 (15)	−0.0039 (11)	0.0117 (12)	−0.0062 (12)
C7	0.0368 (12)	0.0290 (11)	0.0337 (12)	−0.0026 (9)	0.0107 (9)	0.0026 (9)
C8	0.0411 (13)	0.0293 (11)	0.0303 (11)	−0.0013 (9)	0.0088 (9)	0.0012 (8)
C9	0.0297 (11)	0.0280 (11)	0.0287 (11)	−0.0016 (8)	0.0025 (8)	0.0014 (8)
C10	0.0444 (14)	0.0295 (12)	0.0376 (13)	−0.0067 (10)	0.0021 (11)	−0.0005 (10)
C11	0.0440 (15)	0.0395 (15)	0.0552 (17)	−0.0157 (12)	0.0036 (13)	0.0100 (13)
C12	0.0407 (15)	0.0578 (19)	0.0489 (17)	−0.0109 (13)	0.0143 (12)	0.0130 (15)
C13	0.0415 (14)	0.0501 (16)	0.0427 (15)	−0.0046 (12)	0.0178 (12)	0.0006 (12)
C14	0.0272 (10)	0.0321 (11)	0.0326 (11)	−0.0008 (8)	0.0058 (9)	0.0005 (9)
C15	0.064 (2)	0.0396 (15)	0.0548 (18)	−0.0110 (14)	0.0261 (15)	0.0073 (14)
N1	0.0389 (11)	0.0255 (9)	0.0373 (11)	−0.0027 (8)	0.0120 (9)	−0.0033 (8)
O1	0.0433 (11)	0.0543 (13)	0.0750 (17)	0.0013 (10)	0.0278 (11)	−0.0227 (12)
O2	0.0528 (13)	0.0308 (10)	0.0659 (15)	0.0113 (9)	−0.0047 (11)	0.0013 (10)
S1	0.0335 (3)	0.0299 (3)	0.0491 (4)	0.0048 (2)	0.0078 (3)	−0.0079 (3)
I1	0.0954 (2)	0.05448 (17)	0.05233 (17)	0.00090 (11)	0.03859 (15)	−0.00527 (9)

### Geometric parameters (Å, °)

**Table:**

C1—C6	1.380 (4)	C9—C14	1.399 (4)
C1—C2	1.381 (4)	C9—C10	1.399 (3)
C1—S1	1.759 (3)	C10—C11	1.365 (5)
C2—C3	1.386 (6)	C10—H10	0.9300
C2—H2	0.9300	C11—C12	1.379 (5)
C3—C4	1.367 (7)	C11—H11	0.9300
C3—H3	0.9300	C12—C13	1.387 (5)
C4—C5	1.375 (6)	C12—H12	0.9300
C4—H4	0.9300	C13—C14	1.392 (4)
C5—C6	1.384 (5)	C13—H13	0.9300
C5—H5	0.9300	C14—N1	1.411 (3)
C6—H6	0.9300	C15—H15A	0.9600
C7—C8	1.355 (4)	C15—H15B	0.9600
C7—N1	1.420 (3)	C15—H15C	0.9600
C7—C15	1.493 (4)	N1—S1	1.667 (2)
C8—C9	1.421 (4)	O1—S1	1.411 (3)
C8—I1	2.050 (3)	O2—S1	1.416 (3)
			
C6—C1—C2	121.9 (3)	C9—C10—H10	120.7
C6—C1—S1	119.1 (2)	C10—C11—C12	121.0 (3)
C2—C1—S1	119.0 (2)	C10—C11—H11	119.5
C1—C2—C3	118.5 (4)	C12—C11—H11	119.5
C1—C2—H2	120.8	C11—C12—C13	122.0 (3)
C3—C2—H2	120.8	C11—C12—H12	119.0
C4—C3—C2	120.0 (4)	C13—C12—H12	119.0
C4—C3—H3	120.0	C12—C13—C14	117.2 (3)
C2—C3—H3	120.0	C12—C13—H13	121.4
C3—C4—C5	121.1 (3)	C14—C13—H13	121.4
C3—C4—H4	119.4	C13—C14—C9	121.0 (2)
C5—C4—H4	119.4	C13—C14—N1	132.0 (3)
C4—C5—C6	119.9 (4)	C9—C14—N1	107.0 (2)
C4—C5—H5	120.0	C7—C15—H15A	109.5
C6—C5—H5	120.0	C7—C15—H15B	109.5
C1—C6—C5	118.6 (3)	H15A—C15—H15B	109.5
C1—C6—H6	120.7	C7—C15—H15C	109.5
C5—C6—H6	120.7	H15A—C15—H15C	109.5
C8—C7—N1	106.9 (2)	H15B—C15—H15C	109.5
C8—C7—C15	129.1 (3)	C14—N1—C7	108.7 (2)
N1—C7—C15	123.9 (3)	C14—N1—S1	125.89 (19)
C7—C8—C9	110.2 (2)	C7—N1—S1	124.72 (18)
C7—C8—I1	125.8 (2)	O1—S1—O2	120.43 (16)
C9—C8—I1	124.00 (18)	O1—S1—N1	105.47 (13)
C14—C9—C10	120.1 (2)	O2—S1—N1	107.93 (14)
C14—C9—C8	107.1 (2)	O1—S1—C1	109.09 (15)
C10—C9—C8	132.8 (3)	O2—S1—C1	107.83 (14)
C11—C10—C9	118.7 (3)	N1—S1—C1	105.06 (12)
C11—C10—H10	120.7		
			
C6—C1—C2—C3	−0.7 (5)	C8—C9—C14—C13	−179.2 (3)
S1—C1—C2—C3	−178.7 (3)	C10—C9—C14—N1	−177.8 (2)
C1—C2—C3—C4	0.7 (6)	C8—C9—C14—N1	1.6 (3)
C2—C3—C4—C5	0.3 (6)	C13—C14—N1—C7	179.0 (3)
C3—C4—C5—C6	−1.1 (6)	C9—C14—N1—C7	−2.0 (3)
C2—C1—C6—C5	−0.1 (5)	C13—C14—N1—S1	8.1 (4)
S1—C1—C6—C5	177.9 (2)	C9—C14—N1—S1	−172.91 (19)
C4—C5—C6—C1	1.0 (5)	C8—C7—N1—C14	1.6 (3)
N1—C7—C8—C9	−0.5 (3)	C15—C7—N1—C14	−179.7 (3)
C15—C7—C8—C9	−179.2 (3)	C8—C7—N1—S1	172.6 (2)
N1—C7—C8—I1	179.13 (18)	C15—C7—N1—S1	−8.7 (4)
C15—C7—C8—I1	0.5 (5)	C14—N1—S1—O1	−19.0 (3)
C7—C8—C9—C14	−0.7 (3)	C7—N1—S1—O1	171.5 (2)
I1—C8—C9—C14	179.62 (18)	C14—N1—S1—O2	−149.0 (2)
C7—C8—C9—C10	178.6 (3)	C7—N1—S1—O2	41.5 (3)
I1—C8—C9—C10	−1.1 (4)	C14—N1—S1—C1	96.2 (2)
C14—C9—C10—C11	−1.3 (4)	C7—N1—S1—C1	−73.3 (2)
C8—C9—C10—C11	179.5 (3)	C6—C1—S1—O1	−139.6 (2)
C9—C10—C11—C12	0.0 (5)	C2—C1—S1—O1	38.4 (3)
C10—C11—C12—C13	1.3 (5)	C6—C1—S1—O2	−7.2 (3)
C11—C12—C13—C14	−1.1 (5)	C2—C1—S1—O2	170.8 (3)
C12—C13—C14—C9	−0.2 (4)	C6—C1—S1—N1	107.7 (2)
C12—C13—C14—N1	178.7 (3)	C2—C1—S1—N1	−74.2 (3)
C10—C9—C14—C13	1.4 (4)		

### Hydrogen-bond geometry (Å, °)

**Table:**

Cg3 is the centroid of the C9–C14 ring.

**Table:**

*D*—H···*A*	*D*—H	H···*A*	*D*···*A*	*D*—H···*A*
C13—H13···O1	0.93	2.29	2.871 (4)	120
C4—H4···Cg3^i^	0.93	2.65	3.517 (5)	156

Symmetry codes: (i) −*x*+2, −*y*, −*z*+2.
